# 2D Materials (WS_2_, MoS_2_, MoSe_2_) Enhanced Polyacrylamide Gels for Multifunctional Applications

**DOI:** 10.3390/gels8080465

**Published:** 2022-07-25

**Authors:** Bengü Özuğur Uysal, Şeyma Nayır, Melike Açba, Betül Çıtır, Sümeyye Durmaz, Şevval Koçoğlu, Ekrem Yıldız, Önder Pekcan

**Affiliations:** 1 Faculty of Engineering and Natural Sciences, Kadir Has University, Cibali, Fatih, Istanbul 34083, Turkey; nayirs@itu.edu.tr (Ş.N.); 20161704046@stu.khas.edu.tr (M.A.); 20161704081@stu.khas.edu.tr (B.Ç.); 20161709007@stu.khas.edu.tr (S.D.); 20161704072@stu.khas.edu.tr (Ş.K.); 20161701051@stu.khas.edu.tr (E.Y.); pekcan@khas.edu.tr (Ö.P.); 2 Faculty of Science and Letters, Istanbul Technical University, Maslak, Istanbul 34469, Turkey

**Keywords:** TMDs, gelation, optical properties, polyacrylamide, multifunctional composite gels

## Abstract

Multifunctional polymer composite gels have attracted attention because of their high thermal stability, conductivity, mechanical properties, and fast optical response. To enable the simultaneous incorporation of all these different functions into composite gels, the best doping material alternatives are two-dimensional (2D) materials, especially transition metal dichalcogenides (TMD), which have been used in so many applications recently, such as energy storage units, opto-electronic devices and catalysis. They have the capacity to regulate optical, electronic and mechanical properties of basic molecular hydrogels when incorporated into them. In this study, 2D materials (WS_2_, MoS_2_ and MoSe_2_)-doped polyacrylamide (PAAm) gels were prepared via the free radical crosslinking copolymerization technique at room temperature. The gelation process and amount of the gels were investigated depending on the optical properties and band gap energies. Band gap energies of composite gels containing different amounts of TMD were calculated and found to be in the range of 2.48–2.84 eV, which is the characteristic band gap energy range of promising semiconductors. Our results revealed that the microgel growth mechanism and gel point of PAAm composite incorporated with 2D materials can be significantly tailored by the amount of 2D materials. Furthermore, tunable band gap energies of these composite gels are crucial for many applications such as biosensors, cartilage repair, drug delivery, tissue regeneration, wound dressing. Therefore, our study will contribute to the understanding of the correlation between the optical and electronic properties of such composite gels and will help to increase the usage areas so as to obtain multifunctional composite gels.

## 1. Introduction

Multifunctional polymer composite gels have been developed to address a wide variety of applications, from absorption-dominated electromagnetic-interference shielding [1] to tissue engineering [2], and even catalysts and water purification [3] due to the synergistic effect between polymer and new generation additives. To achieve this multifunctional effect, 2D materials are one of the best materials that can be integrated into polymer gels.

Among the family of 2D materials, graphene, and transition metal dichalcogenides (disulphides and selenides of molybdenum and tungsten, etc.) have started to be used in the production of opto-electronic devices and catalysis, and also, they have attracted attention in the field of energy storage units as nano-filler materials [4,5]. Besides the other transition metal dichalcogenide (TMD) materials, molybdenum disulphide (MoS_2_) is the most important one because of its superior electronic behavior and mechanical properties [6,7]. Another representative of this two-dimensional material group is Tungsten disulphide (WS_2_), which can be used for advanced applications such as photovoltaic and photocatalysis due to its size dependent tunable band gap energy. Both MoS_2_ and WS_2_ are classified as semiconductor materials because they both have band gap energies of about 1.2 eV. The indirect bandgap of MoSe_2_ in its bulk form has a value of 1.1 eV. A direct band gap of 1.5 eV can be obtained by exfoliating MoSe_2_ into a few layers [8]. The band gap energy of as-grown MoS_2_ flakes from chemical vapor deposition can be modulated from 1.86 eV to 1.57 eV [9]. Furthermore, it is reported that the band gap energies of TMDs are in the range of 1.5–1.8 eV [10,11,12]. However, the magnitude of this band gap energy is insufficient for many electronic applications [13,14]. In fact, the minimum band gap energy required for technological applications must be greater than 1.8 eV. The band gap energy of 1.8 eV is the limit value that allows one to turn between on and off states through the conduction of MoS_2_ [15]. Two-dimensional MoS_2_ possesses this intrinsic band gap energy that makes it ideal for applications in electronics, optoelectronics and biosensors [16]. Therefore, it is necessary to prepare composites of different materials to provide the desired band gap values of these 2D structures [17].

In addition, it has been thought that the molybdenum dichalcogenides (MoS_2_ and MoSe_2_) have the capacity to increase and regulate mechanical properties when incorporated into basic molecular hydrogels [18,19]. On the other hand, the most common hydrogel is polyacrylamide (PAAm), which is affordable; it can be formed into desired shapes and has the flexibility to match biological materials [20]. It has many realized and potential applications including drug delivery systems and chemo-mechanical devices. PAAm applications have recently moved their focus to using them to create innovative polymer systems with unique structure and functionalities, because PAAm can be chemically infused with other elements or compounds to use in applications of wound dressing, biosensors, drug delivery, tissue regeneration and cartilage repair [21]. The copolymerization process of hydrogel composites made of PAAm and various reinforcing additives is also important for its clinical uses in cell biology and drug delivery applications [22]. Encapsulation of nanoparticles such as silicon, carbon nanotubes, gelatine, cellulose, and other materials improves the strength, bonding, and self-healing properties of the polyacrylamide hydrogel [23,24,25,26,27,28]. PAAm as promising hosting organic matrix for composite materials not only provides mechanical stability, but also new functionalities after the incorporation of conducting materials with different structures.

Similarly, previous studies [29,30,31,32] have shown that different nano-filler materials (carbon nanotube and graphene oxide)-doped polymers such as polyacrylamides (PAAm), latex, polystyrene have also manifested better mechanical and electrical properties. In this work, the amount of dependent electronic and optical properties of MoS_2_, MoSe_2_, WS_2_-doped PAAm was investigated. It is normally accepted that the composite hydrogels can effectively connect the unusual properties of the inorganic (TMD) and organic (PAAm) components to get the desired properties for multifunctional materials.

## 2. Results and Discussion

### 2.1. Gelation Process and Drying of Composite Gels

**Figure 1:**
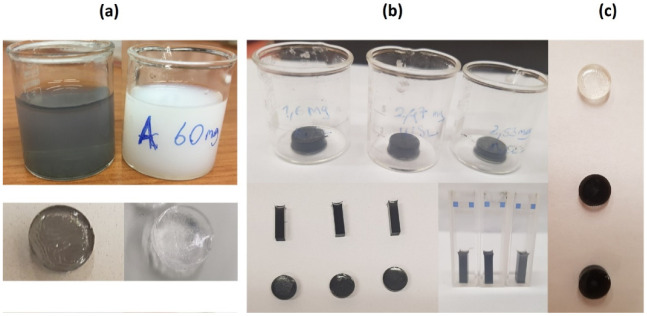
(**a**) **Above:** MoSe_2_-doped PAAm and PAAm just after the gelation. **Below:** The same gels after drying in air. (**b**) **Above:** Disc-shaped samples after drying in air. From left to right, MoS_2_, WS_2_, MoSe_2_-doped PAAm. **Below:** Dry gels in quartz cuvette, and disk-shaped by drying inside the beaker. (**c**) Disk-shaped gels. PAAm at the top, PAAm + 7.5 mg WS_2_ in the middle, PAAm + 10 mg WS_2_ at the bottom.

The gelation process of PAAm takes place very quickly. For this reason, a part of the solution (approximately 3 mL) in the beaker was poured into the quartz cuvette immediately after N,N,N′,N′-Tetramethylethylenediamine (TEMED), which is used as an essential catalyst, was added to the solution and quickly placed in the spectrophotometer cabinet. As a result, composite gels with different TMD amount have the form of a square prism and disc shape as presented in Figure 1.

The AAm solution, which is white at first, loses its opacity and becomes transparent while it is drying. When the effect of drying on the optical clarity of the gels was examined, it was observed that all the gels subjected to the drying process became more transparent. This characteristic of the gel indicates that there are two different regions as polyacrylamide-rich and aqueous inside the gel. Thus, the gels scatter light vigorously as a result of the fluctuations in refractive index of these two regions. This leads to the decrease in transmission of the gel. The gel is opaque. After drying, water content and in other words, the number of aqueous regions in the gel are decreasing. Therefore, the refractive indices of all regions are well matched. There are no significant fluctuations. As a result, gels become transparent. Figure 1a depicts the white AAm solution in the beaker (above right) and its dried gel (below right). One can see that the gel was opaque and white; after drying in air, it became hard, transparent, and shrunk in size. TMD-doped PAAm gels have a grey color and after drying, they have a dark grey color due to the decrease in the water content. Figure 1b shows MoS_2_, WS_2_, MoSe_2_-enhanced PAAm dried gels with different geometrical shapes, from left to right, respectively. For the added TMDs to be compared with each other, they were included in the solution with masses corresponding to the equimolar ratio (the masses are seen on the beakers in the upper figure). After the acrylamide was completely dissolved, TMD was added. A homogeneous solution could not be obtained, since TMDs were gathered and agglomerated in the middle of the gel. Before the insertion of TMD, Polyvinylpyrrolidone (PVP), which is an organic adhesive, was added to the solution so that homogenous and evenly distributed TMD inside the gel was observed.

Many experiments have been carried out in different studies on the interaction of different doping materials with PVP [33,34,35,36] and it has been observed that the polymer composites formed reach their most homogeneous state by adding PVP. This would be due to experimental evidence showing that the use of PVP can significantly speed up the exfoliation of TMDs in composite solutions. This results in a homogenous solution and limits the amount of surface imperfections [37,38]. Figure 1c shows PAAm composite gels containing different amounts of TMD additives.

### 2.2. Optical and Electronic Properties

To describe and understand the gelation process, one can use the following kinetic equation showing the relationship between the rate of monomer consumption, which is the derivative of the concentration of monomer, [*M*] with respect to time, *t*, and the rate of the polymerization [39].

(1) $$d\left[M\right]/dt=-k_{r}\left[M\right]$$

Here, *k_r_* is the constant rate of gelation. If one needs to solve this equation, it yields

(2) $$\left[M\right]=\left[M_{0}\right]\exp\left(-k_{r}t\right)$$

where [*M*_0_] is the concentration of monomer at *t* = 0. During the gelation process, describing the phenomena, Equation (2) is needed. According to Equation (2), the concentration of monomer is decreasing exponentially with time, since the consumption of monomers is creating the microgels that lead to the turbidity. On the other hand, spectroscopic measurements of transmitted light through the gels also allow one to monitor the gelation process. The turbidity in the medium is decreasing and causes a decrease exponentially in the intensity of photon transmission, *I_tr_*.

(3) $$I_{tr}=I_{0}\exp\left(-k_{r}t\right)$$

where *I*_0_ is the intensity of the incident photon at *t* = 0 [40]. The ratio of *I_tr_* and *I*_0_ yields the transmittance of gels.

A slight difference between the light transmission abilities of the PAAm gels doped with different TMDs is observed in Figure 1b. Spectrophotometric measurements are required to distinguish them in terms of light transmittance. After pouring the solution into the quartz cuvette, the transmittance percentages of the solutions before, during, and after gelation were measured with a uv-vis spectrophotometer.

**Figure 2:**
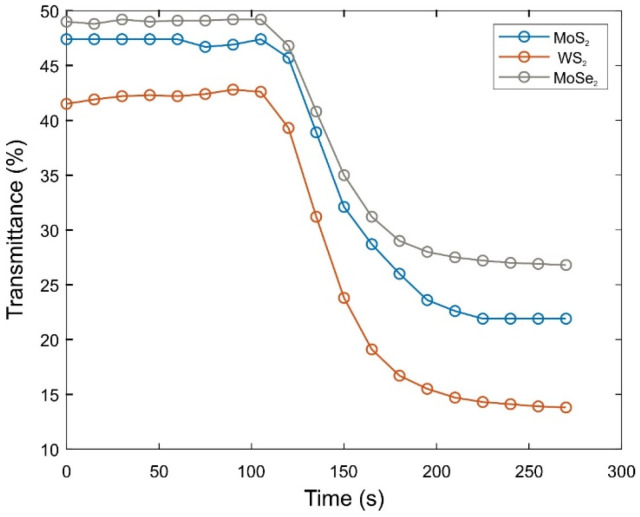
Transmittance versus gelation time graph of 2D materials-doped PAAm gels.

The transmittance of the TMD-doped PAAm composites are presented in Figure 2. It is seen that the intensity decreased dramatically above a certain time indicating that opalescence occurs during gelation for each composite gels.

**Figure 3:**
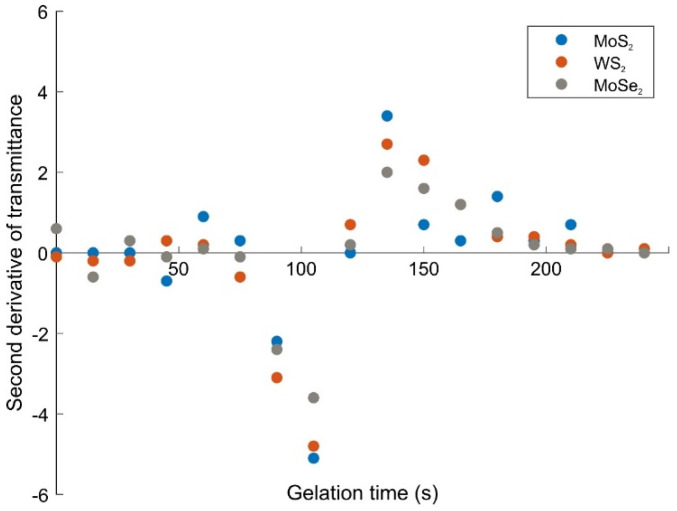
Second derivative of transmittance of the TMD-doped PAAm gels with respect to time.

The statistical models have been shown to approximate the results obtained by Tanaka and new effects derived from the Flory–Stockmayer theory pointing to the solution of the site–link correlated percolation problem [41,42,43,44]. According to the assumptions, before and after the sol–gel phase transition points, the trends can be different. The curve resembles a sigmoidal and it fits the Boltzmann Sigmoidal Function [45,46,47,48]. These curves were used to determine gel points. According to the nature of them, the second derivative of the transmittance with respect to time must be zero around the gel point. The second derivative of the transmittance with time is shown in Figure 3. After 120 s, the curves change the trend from negative to positive. Hence, the gel point of the composite gels around 120 s. This result is important for hydrogel studies accompanying all biological target materials, since the delivery of hydrogels to the body must occur while they are in liquid form (just at the right time). Afterwards, the hydrogel should turn into a gel inside the body.

**Figure 4:**
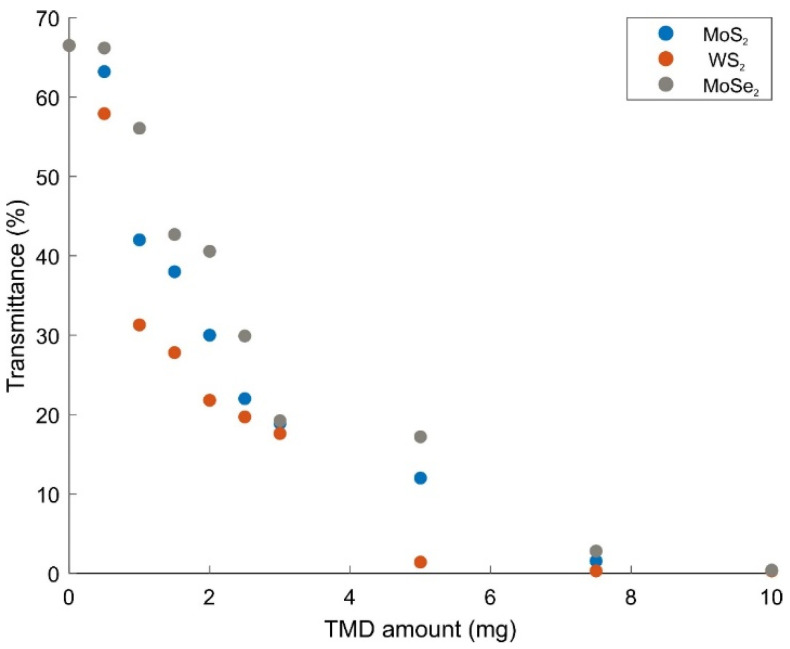
Transmittance plot of PAAm gels reinforced with MoS_2_, WS_2_ and MoSe_2_ depending on the amount of TMD.

All composite gels were allowed to dry at room temperature. The relationship between the amount of TMDs and the transmittance response of the composite gels to the incident photon is shown in Figure 4. A decrease in transmittance due to WS_2_ content occurs. The change in the color of the gel and its gradual darkening from clear to black are shown in Figure 1c. This observation is in agreement with the change in transmittance in Figure 4. Both MoS_2_ and MoSe_2_-enhanced PAAm composite gels have also the same trendline. Transmittance of MoSe_2_–PAAm composites with various MoSe_2_ amounts measured using a uv-vis spectrophotometer is inversely proportional to the MoSe_2_ amount as expected. For MoS_2_-enhanced PAAm composite gels, when the light transmission behavior is examined, a decrease in the form of exponential decay of transmittance is observed as the amount of MoS_2_ increases. If these gels are to be used for a multifunctional application, it will not be sufficient to have only transparent, only conductive, or only elastic property. In order to obtain a composite gel that combines as many properties as possible, those with an average transmittance value can be taken. Therefore, if the transmittance values of these gels are 20% and below, they should be eliminated. The absorbance curves of the composite gels prepared with the remaining TMD amounts were evaluated to calculate the band gap energy.

**Figure 5:**
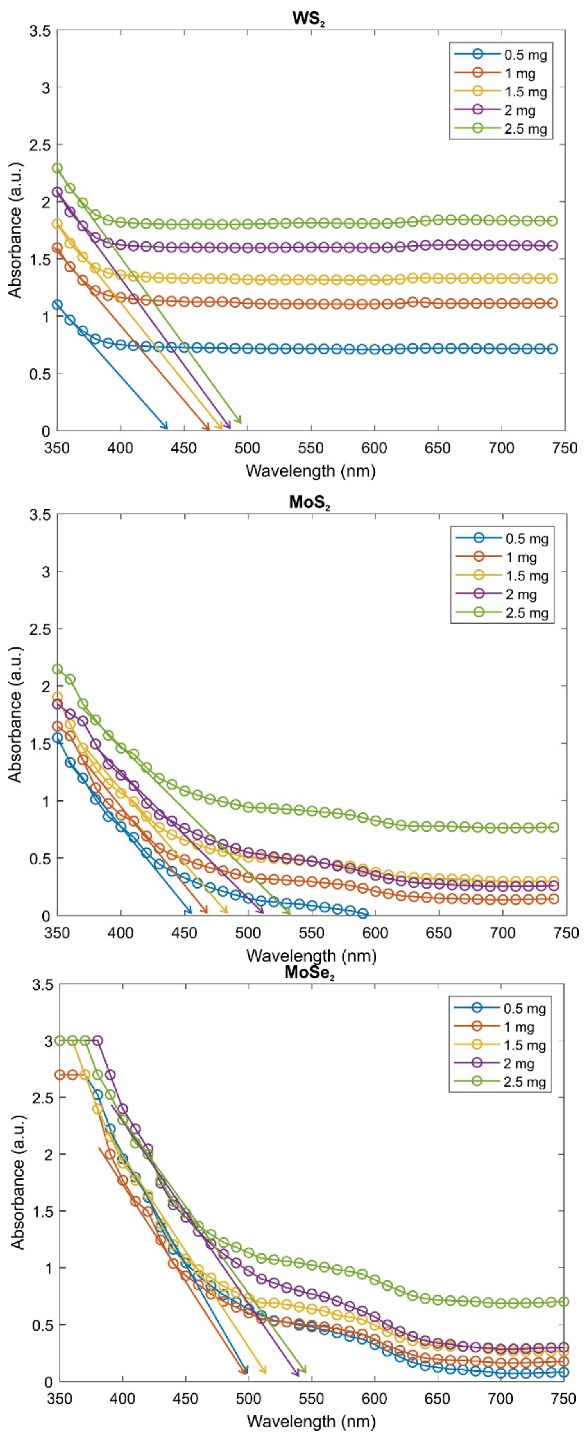
Absorbance versus wavelength graph of WS_2_, MoS_2_, MoSe_2_ reinforced PAAm composites from top to bottom, respectively.

After drying in air, the absorption response of composite gels was measured by a uv-vis spectrophotometer. Figure 5 represents absorbance versus wavelength curves for all TMDs (WS_2_, MoS_2_, MoSe_2_) with various TMD amounts. It was found that the absorption edge shifts to a shorter wavelength with the decreasing TMD amount.

Just like in this study, it is not known what kind of interband transition (direct, indirect, allowed or forbidden) of electrons from the occupied state in the conductive state to the empty state in the valence band of composite materials. In this case, it will be difficult to use the Tauc model [49] when calculating the band gap energy values of composite materials.

Therefore, researchers have tried different methods. The most striking of these in recent years is the dielectric loss method [50]. The band transition of electrons is related to the electron–photon interaction. The imaginary part of the dielectric function which corresponds to the dielectric loss describes the interband transition type. This new method can be used to estimate the band gap energy of composites with high accuracy. However, this requires a long and difficult calculation including the extinction coefficient and refractive index [51,52]. On the other hand, the Tauc model is a reliable method to specify the type of electronic transition.

In addition to this information, the band gap energy values at the absorbance edge can be calculated in order to have at least an idea about the band gap energy values of the new TMD-enhanced PAAm composite gels to determine the ones prepared with TMD in a sufficient amount for multifunctional applications. Since the grain size of at least WS_2_ is roughly 1 micrometer, it would be accurate to discuss grains considerably larger than nanometers when the polymer composite is obtained, and therefore, examining the shift of the absorbance edge has to be taken into account. In this case, as the amount of TMD increases, the extrapolated wavelength value of the composite, expressed as the absorbance edge, shifts to larger wavelengths.

The extrapolation of the absorbance curve with respect to the wavelength (arrows in Figure 5) gives the band gap energy at the absorption edge. These energy values are given in Table 1, after calculating from the following formula using the wavelength values at which it cuts the curve. The Planck–Einstein equation between photon energy, *E*, and wavelength, λ, is:

(4) E=hc/λ

Here, *h* is the Planck constant, and *c* is the speed of light. The increase in TMD amount occurs with a red shift in the absorbance spectrum of composite gels. The absorbance intensities also become strong with an increment in the TMD amount inside the composite gels. The band gap energy at the absorption edge decreases with an increase of the values of TMD amount as expected.

The band gap energy detected from the absorption edge is usually found to be smaller than the energy calculated from the Tauc plot [49]. Considering this, it would be appropriate to prefer composites with a band gap of around 2.7 eV, which is close to the energy value range required for biomedical applications [53]. On the other hand, for electronic applications [54], those with a higher TMD content of around 2.4 eV should be chosen, or if the composite is considered to be used as a transparent conductor material, the composite with the highest transmittance and around 2.8 eV of band gap energy can be selected. When one compares the values in Table 1, one can consider the 2 mg TMD added PAAm composite gel, which has average optical and electronic properties, as the ideal gel after fine tuning. It can be subjected to further investigations regarding the calculation of band gap energy. The absorption coefficients, α, can be calculated by the equation:

α(hν) = (log_e_10/t) A(hν) (5)

where t is the thickness of the discs, and A is the absorbance for each photon energy. Then, the band gap energy was calculated from the Tauc’s plot by varying the absorbance values with respect to the wavelength for each composite gel.

The band gap energy (E_g_) between the maximum of the conduction band and the minimum of the valence band of an amorphous semiconductor is determined by Tauc [49] in 1966. The following can be written for direct transition:

(αhν)^2^ = C (hν − E_g_) (6)

**Figure 6:**
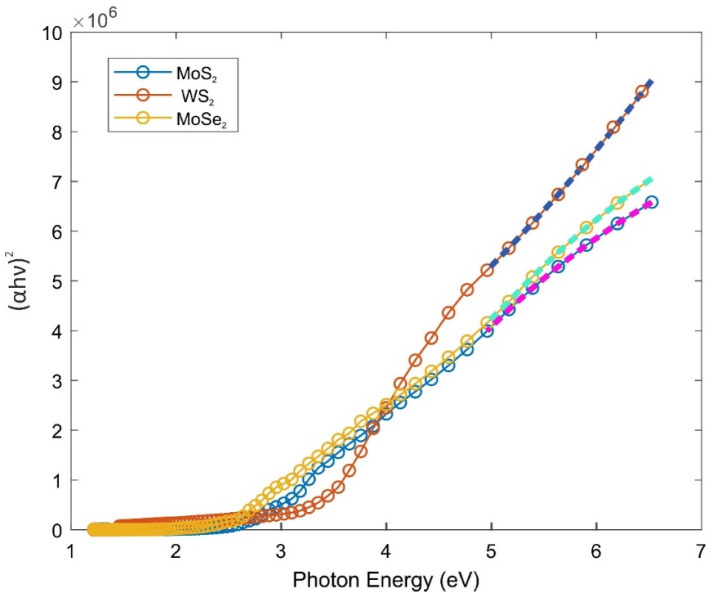
Tauc plots of MoS_2_, WS_2_, MoSe_2_ enhanced PAAm composite gels. The linear fitting equations given in Table 2 were used to calculate band gap energies of these composite gels.

The value of the crossing photon energy is taken as the band gap energy. A characteristic Tauc plot of the WS_2_/MoS_2_/MoSe_2_ + PAAm composite gel with 2 mg of TMD doping is shown in Figure 6. The band gap energies were obtained by extrapolating the (αhν)^2^ linear fit. The linear fitting equations and their R^2^ values are given in Table 2. The reason why composite gels were taken as direct band transition is that TMDs in the form of 2D sheets are exfoliated as separate layers [8,9] in the PAAm gel matrix.

Narrow or lower band gap energy is attributed to the high electrical conductivity of the material [55]. Therefore, the increase in the TMD amount leads to the decrease in band gap energy that corresponds to the increase in conductivity. Band gap energies were calculated and found in the range 2.48–2.84 eV. Table 2 summarizes these results for different composite gels.

The values of band gap energy have been found to be in the range of 4.70–5.10 eV for PVP-reinforced PAAm composites [56]. On the other hand, the band gap values calculated for MoS_2_/PVP/PAAm composites with different amounts of MoS_2_ were found in the range of 3.10–3.81 eV [57]. The estimated band gap energies of WS_2_/PVP/PAAm composites with various WS_2_ contents were between 3.35 and 4.57 eV [58]. It is observed that the band gap energies of the composites are in good agreement with the previous results. Since the amounts of Bis and PVP used to prepare composites in previous studies were different from the current study, the band gap energy value ranges found were different. By changing the amount of Bis and PVP, band gap energy can be regulated in accordance with the desired optical and electronic properties for multifunctional applications. Using this information, another group of researchers can easily obtain the composite gel by determining a special TMD amount according to the desired band gap energy and transmittance combination for the specific application.

## 3. Conclusions

Characteristics of polymer-based nanocomposites continue to be an attractive area of interest for materials scientists. This study aims to reveal the relationship between tunable band gap energy and optical properties of 2D materials-doped polyacrylamide composite gels with the effect of the TMD amount. MoS_2_, MoSe_2_, WS_2_-doped PAAm composites were prepared using free-radical polymerization. The optical properties of MoS_2_, MoSe_2_, WS_2_-doped PAAm were investigated. An increment of the TMD amount caused a decrease in the compactness of the composite’s macrogel structure.

The properties of gels can be controlled by changing TMD content. The results indicate that an increase in the amount of TMD leads to a decrease in the gel points. The calculated band gap energies of the composites are in harmony with the literature. The optical results indicated that the absorption edge shifts to a shorter wavelength with the decreasing TMD amount. The properties of the final gels are highly dependent on the amount of TMD, Bis, and PVP present during the formation of gels. Composite gels with high TMD content exhibit stronger absorption in the longer wavelength region compared to gels with low TMD content, which consequently decreases the band gap values of the films. In this case, it can be concluded that composite gels are better semiconductors regarding the conductivity if the correlation between conductivity and band gap energy is considered. We have chosen the 2 mg TMD-added PAAm composite gel, which has average optical and electronic properties, as the ideal gel after fine tuning. The band gap energy of the films was found to be in the range between 2.48 and 2.84 eV. This range fits the characteristic band gap energy range of promising semiconductors. It is observed that the band gap energies of the composites are in good agreement with the results of the previous studies on TMD-doped PAAm. Consequently, the optical and electronic properties of these gels can be altered by the change in the amount of TMD of the composite gels. Due to these characteristics, these composites can be used in electronic and optical applications in addition to biomedical applications. It has been determined by previous studies in the literature that according to the mechanical properties of TMDs, enhanced PAAm gels have already been registered as flexible materials. Optical and electronic properties of these new composite gels have been studied so that they can be used in multifunctional applications. The approach used in this study is promising in modulating the optical and electronic properties of other transition metal dichalcogenide monolayers. This study reveals that the wide optical band gap of composite gels can be significantly tuned by incorporating 2D structures. As future work, the surface resistivity and the conductivity of these composite gels can be investigated using AC current-voltage measurements that are crucial for the electronic applications.

## 4. Materials and Methods

### 4.1. Preparation of WS_2_, MoS_2_, and MoSe-Doped PAAm Composite Gels

Solutions were prepared via free radical crosslinking copolymerization technique at controlled room temperature (22.8–23.1 C) and ambient relative humidity (32–38%) by solving Acrylamide (AAm, Merck, Rahway, NJ, USA), Polyvinylpyrrolidone (PVP, Sigma-Aldrich, Saint Louis, MO, USA), N,N′-Methylenebisacrylamide (BIS, Sigma-Aldrich), Ammonium persulfate (APS, Sigma-Aldrich), and N,N,N′,N′-Tetramethylethylenediamine (TEMED, Sigma-Aldrich), respectively, in distilled water by stirring magnetically and mechanically. This experimental technique is a similar procedure mentioned before in our previous works with different BIS and PVP amounts [48]. Distilled water prepared by the Chemistry Laboratory of KHU was used as the reaction solvent. In order to provide various composite solutions and compare the characteristics of them, TMD content with the same molar value for WS_2_, MoS_2_, and MoSe_2_ was added to the solution after Acrylamide and PVP were completely dissolved in distilled water. The whole preparation process was repeated by changing only the amount of TMD so that the amounts of the other chemical contents were kept the same, and composite gels containing different amounts of TMD were prepared.

### 4.2. Characterization

The gelation of Acrylamide with various amounts of TMD was measured using a Labomed Spectro 22 uv-vis Spectrophotometer. The change in the photon transmission during the gelation of Acrylamide with various amounts of WS_2_, MoS_2,_ and MoSe_2_ was measured at λ = 650 nm wavelength. The reason why all transmission measurements were performed at a wavelength of 650 nm, is one of the maxima wavelengths of the halogen lamp inside the spectrophotometer. Gelation process and transmittance of photon were monitored in real-time by the camera system. Gel points of the polymer composites, t_gel_, were obtained. Absorbance and transmittance measurements were taken for single wavelength one by one. Absorbance and transmittance were noted after the calibration of the spectrophotometer for each wavelength.

## Figures and Tables

**Table 1:** The calculated band gap energies of all composites at the absorbance edge.

TMD Amount in WS_2_/MoS_2_/MoSe_2_ + PAAm Composite Gel (mg)	Bandgap Energy of WS_2_ + PAAm (eV, from Absorbance Edge)	Bandgap Energy of MoS_2_ + PAAm (eV, from Absorbance Edge)	Bandgap Energy of MoSe_2_ + PAAm (eV, from Absorbance Edge)
0.5	2.91	2.73	2.56
1	2.73	2.66	2.53
1.5	2.69	2.55	2.49
2	2.59	2.40	2.46
2.5	2.50	2.35	2.43

**Table 2:** The calculated band gap energies of all composites from the Tauc plots.

Type of the Composite Gel	Linear Fitting Equations with R^2^ Values	Bandgap Energy (eV, from Tauc Plot)
MoS_2_ + PAAm	y = (1.650 × 10^6^)x − 4.087 × 10^6^ R^2^ = 0.994	2.48
WS_2_ + PAAm	y = (2.434 × 10^6^)x − 6.923 × 10^6^ R^2^ = 0.998	2.84
MoSe_2_ + PAAm	y = (1.867 × 10^6^)x − 5.028 × 10^6^ R^2^ = 0.996	2.69

## Data Availability

The data that support findings of this study are available from the corresponding author upon reasonable request.
